# 肺部转移性肿瘤的临床诊治策略

**DOI:** 10.3779/j.issn.1009-3419.2014.03.18

**Published:** 2014-03-20

**Affiliations:** 200030 上海，上海交通大学附属胸科医院，上海市肺部肿瘤临床医学中心胸外科 Department of Thoracic Surgery, Shanghai Chest Hospital/Shanghai Lung Tumor Clinical Medical Center, Shanghai 200030, China

肺是所有恶性肿瘤最主要的转移靶器官，约30%-40%的恶性肿瘤在自然病程中发生肺转移^[1, 2]^。据尸检报告^[3]^，几乎1/3死于癌症的患者伴有肺转移。肺转移常提示病情进入晚期，若未经及时发现和治疗，可致患者病情急转直下，严重者迅速死亡。目前，肺转移瘤的研究是一大热点和难点，本文拟就其临床特点和诊治策略作一系统综述。

## 1 肺转移瘤的流行病学及病因机理

人体不同部位不同病理类型的恶性肿瘤，均可以转移到肺部。据中国医学科学院肿瘤医院10年间（1999年-2009年）3, 569例肺转移肿瘤原发部位的调查分析，原发于肺以外肿瘤出现肺转移最多的是乳腺癌（16.92%），其次为大肠癌（15.86%）、甲状腺癌（7.68%）、肝肿瘤（7.48%）、淋巴瘤（6.61%）、肾癌（6.39%）、食道肿瘤（6.08%）和子宫肿瘤（5.41%）等。肺之所以成为全身转移性肿瘤发生率最高的脏器，主要是由于肺是体循环的第一过滤器，全身血流的必经之路^[4]^；其次，肺循环是低压系统，血流缓慢，肿瘤细胞易于停滞；加之肺接受肺动脉和支气管动脉的双重血管供应，血供营养丰富，使肿瘤细胞的肺部转移几率增大。

## 2 肺转移的途径

肿瘤转移是一个多环节、多阶段的过程。依原发肿瘤不同，转移途径各异，可分为血行转移、淋巴转移、直接蔓延、气道转移等。①血行转移：最多见，肺为全身血液的中间过滤站，全身血液流经肺毛细血管网循环，脱落游离于血液中的肿瘤细胞极易停留于肺部生长，形成转移灶。因重力影响和肺底部血流量偏多，转移灶出现在中下肺野的概率比上肺野明显增多。②淋巴转移：多数肿瘤细胞经胸导管回流到体静脉，少数转移至纵隔淋巴结并经淋巴管逆流到肺，瘤细胞浸润引起肺间质增厚，表现为淋巴管炎型或肺门纵隔淋巴结肿大。淋巴转移灶的原发肿瘤多数来源于消化道、肺、乳腺及女性生殖系统的恶性肿瘤。③直接浸润：多为邻近病变的直接侵犯，其原发病变主要来自乳腺、肺及消化道恶性肿瘤。④气道播散转移：少见，支气管肺泡癌可出现气道播散。

## 3 肺转移瘤的临床特点

除原发肿瘤引起的相关症状外，大多数肺转移瘤早期没有明显的特殊临床症状，尤其是血行转移者。一般在随访原发肿瘤的过程中，进行胸部影像检查始被发现。后期可有胸痛、胸闷、咳嗽咳痰、咯血、低热、气急及消瘦等症状，部分患者可并发肺炎。当肺组织被大块肿瘤挤压、气道梗阻或胸腔积液则会出现呼吸困难。对无临床症状的肺转移瘤应该联想到一些较为隐蔽部位的肿瘤，如胰腺癌或胆道肿瘤。特殊患者，如有癌性淋巴管炎者会出现较重的呼吸困难和干咳；气管内转移瘤常出现喘鸣或咳血；胸膜转移者可导致转移部位胸痛；肺尖转移可出现Pancoast综合征。

## 4 肺转移瘤的诊断

根据肺部X线和胸部CT表现，结合原发肿瘤的诊断或病史，一般不难诊断肺转移瘤。也有部分患者先发现肺部结节，继而才寻及原发肿瘤。多发肺转移瘤CT扫描较可靠，可以检出小于1 cm的病灶。对于未能找出原发灶的转移性肺肿瘤，包括甲状腺转录因子-1（thyoid transciiption factor-1, TTF-1）、雌激素受体（estrogen receptor, ER）、前列腺特异性抗原（prostate specific antigen, PSA）、巨囊性病液状蛋白（gross cystic disease fluid protein, GCDFP）、抑制素B（inhibin B, INHB）、肝细胞相关抗原（hepatocellular carcinoma related antigen, HEP）和肾细胞癌单克隆抗原（renalcellcarcmoma monoclonal antigen, RCC Ma）等免疫组化有助于鉴别诊断原发灶。X线表现：转移瘤好发于中下肺野，瘤灶多边缘光滑锐利。部分肝源性肺转移瘤病例表现为右膈抬高或局限性隆起，这可能是肝癌肿大或癌肿向膈面侵袭生长所致^[5]^。临床实践中，对肺部出现单发或多发性大小不等的结节病变，临床上既无症状又无原发恶性肿瘤病史，用肺部其它病变难以解释者，需考虑肺转移瘤。CT表现：主要表现为肺内单发或多发球形结节影，大小不一，边缘光滑，密度均匀，多分布于肺外围。也可出现两肺满布的粟粒样结节。随访过程中可发现转移灶呈进行性增大。PET表现：使用PET检查肺转移肿瘤，敏感性和特异性均高于CT。PET可以发现87%的肺转移病灶。对肺门、纵隔淋巴结转移PET也较CT具有优势。但PET检测直径小于1 cm肺转移病灶时敏感性不足，因此对于病灶较小者推荐结合PET和薄层CT。

## 5 肺转移瘤的治疗

### 5.1 非手术治疗

#### 5.1.1 放射治疗

外放射包括三维适形放疗及螺旋断层放疗。目前三维适形放疗应用较为广泛，能够有效地控制肺转移灶，延长患者的生存时间，且不良反应小。另外，螺旋断层放疗利用螺旋CT成像的逆原理进行放射治疗，原则上可以在人体内实现各种要求的剂量分布。其最大的优点是可同时照射多靶区，相比常规放疗和常规调强，可以实现适形度高得多的剂量分布，可比拟质子治疗效果。Jang等^[6]^报道用螺旋断层放疗机，对42例肝癌肺转移患者的103个肺转移灶进行放疗，肺内病灶完全缓解达到26.3%。内放射治疗则是经CT引导下经皮穿刺植入放射性粒子，如I^125^等。这些放射性粒子均具有在肿瘤靶点短距离内剂量迅速衰减的特点，可使治疗靶点剂量很高，而周围正常组织受照射量极低，从而达到最大程度地消灭肿瘤和保护正常组织之目的。此方法适用于周围型肺转移瘤病灶，同时可作为一线化疗或动脉栓塞化疗等疗法的补充。对于肺转移灶较大，伴有癌性胸水、肺不张、纵隔淋巴结转移的患者，如果外放射治疗对邻近重要脏器如心脏、大血管及食管等的影响较大时^[7]^，可考虑应用内放射。

#### 5.1.2 无水乙醇注射治疗

在CT引导下行经皮肺瘤内无水乙醇注射治疗，无水乙醇可引起肿瘤组织凝固性坏死，并可使肿瘤周围血管上皮细胞坏死，形成血栓，进一步促进肿瘤组织缺血坏死^[8]^。顾仰葵等^[9]^报道，17例肝癌肺转移患者，经CT引导下行经皮肺瘤内无水乙醇注射治疗，此17例患者的37个肺内转移灶中31个病灶无增大，其中26个病灶瘤内乙醇沉积完全，5个病灶沉积良好；注射后8周疗效评价有效率达到83.8%，1年生存率64.2%。该方法创伤小、疗效确切、并发症少，值得临床推广应用。

#### 5.1.3 经皮射频消融治疗

利用射频电磁波在生物介质中产生的热及非热效应凝固癌组织，杀死癌细胞。肿瘤细胞对热的耐受能力比正常细胞差，49 ℃以上即可发生不可逆的细胞损伤。射频电极发出高频率射频波，所产生的热量可使局部温度达到90 ℃以上，能快速有效地杀死肿瘤细胞。Dupuy等^[10]^首次报道了使用射频消融（radiofrequency ablation, RFA）治疗肺部肿瘤，共3例患者（2例原发性肺癌，1例肺转移瘤），3例患者均无气胸等并发症，转移瘤患者6周后CT随访显示经射频消融后，病灶较消融前缩小明显，由此提出肺部肿瘤应用RFA治疗是一种安全可靠的治疗手段。另外，Hiraki等^[11]^对32例肝癌患者的83个肺转移瘤病灶行CT引导下射频消融治疗，手术成功率达到100%，有效率92%，术后3年生存率达57%，证明射频消融术是治疗肺转移瘤的安全有效方法之一。

#### 5.1.4 化疗

多数肺转移瘤对化疗的敏感性和原发灶一致。对化疗敏感的肿瘤出现肺转移，特别是双肺多发转移者，根据原发疾病的方案化疗是一个合理的选择。孙晓非等^[12]^报道了44例青少年恶性生殖细胞瘤患者经化疗后3年总生存率为84.8%，其中1例合并肺转移患者化疗后获得完全缓解。Verma等^[13]^认为，乳腺癌肺转移患者选择蒽环类、紫杉醇类等系统化疗，有效率可达40%。由此可见，对化疗药物敏感的生殖细胞瘤、乳腺癌等化疗后常可取得良好的疗效甚至治愈^[14]^。但有些肿瘤，如恶性黑色素瘤肺转移，化疗常常无明显效果。Barth等^[15]^报道，早期恶性黑色素瘤患者行根治性切除预后较好，一旦发生远处转移，预后很差，中位生存期仅6个月-8个月，5年生存率5%-6%，化疗并不明显改善预后，此时，生物治疗可作为恶性黑色素瘤肺转移患者治疗的选择^[16]^。

#### 5.1.5 靶向治疗

分子靶向治疗主要是通过特异性阻断肿瘤细胞的信号转导或阻止肿瘤血管生成的潜在靶点以抑制肿瘤细胞的生长和增殖。如*EGFR*基因突变的非小细胞肺癌出现肺转移，且在一线或二线治疗失败后可以选择酪氨酸激酶抑制剂吉非替尼或厄洛替尼进行治疗^[17]^；胃肠间质细胞瘤出现肺转移也可以用相应分子靶向治疗药物舒马替尼^[18]^；肝细胞癌、肾透明细胞癌出现广泛的肺转移，如果不适合手术切除也可用索拉非尼治疗^[19]^；乳腺癌的分子靶向治疗等已广为人知。Kudo等^[20]^报道了15例肺转移晚期肝癌的患者服用靶向药物索拉菲尼后完全缓解，并预计索拉菲尼联合切除、消融、栓塞等治疗将明显延长中晚期肝癌患者的生存期，提高生活质量。随着基础研究和临床实践的深入，分子靶向治疗的疗效有望进一步得到提高。

其次，Thaker等^[21]^报道，慢性疲劳和抑郁可通过影响神经、内分泌和免疫系统，促进肿瘤生长和血管生长，提示注意休息，保持身心愉悦的重要性。必要时可考虑中医中药治疗以增强机体免疫功能，也或是肺转移瘤的辅助治疗措施之一。

### 5.2 肺转移瘤的手术治疗

以往人们认为恶性肿瘤有了远处转移即为手术禁忌，然而许多学者对这些“禁区”进行了尝试^[22, 23]^。首例肺转移瘤切除1927年由Divis在欧洲报道，美国Barney和Churchill在1939年对肾癌肺转移的患者进行了肺转移瘤的手术切除，结果患者在术后存活了23年。目前，一系列回顾性研究^[24, 25]^表明，对符合适应证的肺转移瘤患者积极进行手术治疗，可获得满意的生存期延长。经过长期的临床实践和研究，逐渐形成了关于肺转移瘤外科治疗手术指证的共识：①原发肿瘤已控制或者可控制；②在保证足够余肺功能的前提下，肺转移瘤能被完全切除；③无胸腔外转移；④患者一般情况和心肺功能等可耐受手术。随着对肿瘤生物学特点认识的发展和微创技术的进步，手术适应证在不断变化。随着化疗及全身免疫治疗的不断进步，手术指征也在相应扩大。回顾性的研究^[26-28]^表明，一些可手术的乳腺癌肺转移患者，手术后5年生存率可达50%，效果优于化疗。Zabaleta等^[29]^对146例肺转移瘤患者进行手术，3年和5年生存率分别为67.4%和52.4%，中位生存期为56个月，提示手术治疗肺转移瘤的重要意义。国际肺转移瘤登记组织（International Registry of Lung Metastases, IRLM）^[30]^对欧美18个中心共5, 206例肺转移瘤切除的回顾性分析表明，生存率最重要的决定因素是转移瘤的可切除性：完全切除者5年生存率为36%，不完全切除者仅为13%；完全切除的病例中有53%出现再发，再次接受转移癌切除者预后优于未再手术者；Koong等^[31]^对于全肺切除治疗肺转移瘤进行了研究，133例第一次治疗肺转移瘤即采用全肺切除的患者，根治组5年生存率达20%，而不完全切除生存未超过2年者，在肺转移瘤术后再次复发而行全肺切除的患者中，5年生存率达30%，未达到根治者5年生存率为0，因而认为长期生存主要取决于能否完全切除转移灶有关。关于切除范围，Pfannschmidt等^[32]^系统性回顾了20个结肠癌肺转移的研究，发现切除范围不是生存时间的影响因素，支持进行有限的肺切除，在切缘阴性的同时尽量保存肺功能，对肺转移瘤不适宜做全肺切除^[33]^。而IRLM^[30]^的5, 206例资料中，行楔形切除、肺段切除、肺叶或双肺叶切除、全肺切除的比例分别是67%、9%、21%和3%，楔形切除可达到与解剖性切除相同的效果。原发灶组织学类型影响预后方面，Todd^[34]^总结了肺转移瘤患者手术切除转移灶后的5年生存率分别为：骨肉瘤20%-50%，妇科肿瘤42%-53.3%，软组织肉瘤18%-28%，肾脏肿瘤24%-53.8%，头颈部肿瘤40.9%-47%，结肠癌21%-38.6%，睾丸癌（3年生存率）51%-71%，乳腺癌31%-49.5%，黑色素瘤0-33%，提示生殖系统肿瘤预后最好，其次是上皮来源的肿瘤，在上皮来源的肿瘤中乳腺癌预后较好，黑色素瘤及软组织肉瘤预后最差。此观点得到了多数学者^[35, 36]^的支持。

## 6 小结

CT及核素显像技术在肺转移瘤的早期诊断中发挥了重要作用。适宜的治疗有益于延长患者生存期和生活质量，但是目前尚无统一的规范，须针对每一个体采取合理有效的治疗方案。在严格指征的前提下，手术有时可获得良好的疗效，值得高度重视并加强筛选。非手术治疗方法纷繁，须针对患者具体情况取舍，并有待临床逐步积累探索。同时，摸索更为合理有效的多学科综合治疗方案亦是重要的研究课题。
